# Calf circumference as a surrogate indicator for detecting low muscle mass in hospitalized geriatric patients

**DOI:** 10.1007/s40520-024-02694-x

**Published:** 2024-02-07

**Authors:** Caroline M. Kiss, Dominic Bertschi, Nadine Beerli, Manfred Berres, Reto W. Kressig, Andreas M. Fischer

**Affiliations:** 1 University Department of Geriatric Medicine FELIX PLATTER, Basel, Switzerland; 2 Institute of Nursing Science, Basel, Switzerland; 3 Department of Mathematics and Technology, University of Applied Sciences Koblenz, Koblenz, Germany; 4 University of Basel, Basel, Switzerland; 5 Present Address: Department of Geriatrics, Inselspital, Bern University Hospital, University of Bern, Bern, Switzerland

**Keywords:** Sarcopenia, Calf circumference, Geriatrics, Older adults, EWGSOP2

## Abstract

**Background:**

Sarcopenia is characterized by low muscle strength, decreased muscle mass, and decline in physical performance. While the measurements of muscle strength and physical performance are easy to perform, an accurate evaluation of muscle mass is technically more demanding. We therefore evaluated the suitability of calf circumference (CC) as a clinical indicator for muscle mass.

**Methods:**

In a cross-sectional single-centre study, geriatric inpatients were assessed for sarcopenia according to the European Working Group on Sarcopenia in Older People 2 (EWGSOP2) consensus. Calf circumference was tested for correlation with appendicular skeletal muscle mass index (ASMI). Receiver operating characteristic curves (ROC) were used to calculate the discriminatory value of the CC cut-off values to differentiate patients above and below ASMI cut-offs for sarcopenia.

**Results:**

In this study population (n = 305, age 83.5 ± 7.0 years, BMI 25.7 kg/m^2^, 65.6% female), the prevalence of sarcopenia was 22.6%. In subjects with low ASMI, mean CC was 29.5 ± 3.4 cm for females and 32.0 ± 3.4 cm for males. A positive relationship between CC and ASMI was found. The optimized cut-off value for CC to identify patients with low ASMI was <31.5 cm for females (sensitivity 78%, specificity 79%), and <33.5 cm for males (sensitivity 71%, specificity 62%).

**Conclusion:**

In clinical settings where imaging technology for muscle mass quantification is not available, simple calf circumference measurement may be used as a dependable indicator for low muscle mass in older adults.

## Introduction

Accelerated and age-related muscle loss leads to sarcopenia and represents one of the major public health challenges among older adults [1]. The consequences of sarcopenia for patients are reduced functionality as well as a high risk of frailty and falls, which are associated with increased morbidity, mortality, higher healthcare costs as well as decrease in quality of life [2, 3]. These facts underline the vast importance of early diagnosis of sarcopenia in order to timely initiate effective therapy options for geriatric patients.

Both, the European Working Group on Sarcopenia in Older People 2 (EWGSOP2) and the Asian Working Group for Sarcopenia (AWGS) define sarcopenia as the combined loss of strength, skeletal muscle mass, and function [1, 4]. In contrast, the Sarcopenia Definition and Outcome Consortium (SDOC) base their diagnosis on low muscle strength combined with low gait speed [5]. While examinations of muscle strength are easier to perform in everyday clinical practice, it is more challenging to measure muscle mass [6]. With regard to sarcopenia diagnostics, the current EWGSOP2 guidelines refer to different options for the quantitative assessment of muscle mass. This includes Dual X-Ray Absorptiometry (DXA), which is, however, only available in specialized facilities. Furthermore, DXA results show inconsistent correlations with decline in muscle strength [7]. Therefore, the Bioelectrical Impedance Analysis (BIA) provides a simple alternative for the quantification of muscle mass. This portable device is more readily available and applicable at a lower cost. Nevertheless, BIA tends to overestimate muscle mass and is susceptible to the hydration status of an individual. Muscle quantification by cross-sectional imaging such as Magnet Resonance Imaging (MRI) and computer tomography (CT) appears to be more precise [8]. While the determination of total body muscle mass via CT is associated with a not insignificant radiation exposure, both methods, CT as well as MRI, are cost-intensive. The mentioned different diagnostic method modalities also provide inconsistent results in terms of muscle quantification and are not directly comparable with each other. This circumstance contributes to the fact that the assessment of muscle mass plays a rather subordinate role in clinical practice, although muscle mass quantification is necessary according to the EWGSOP2 and AWGS guidelines in order to define and establish the diagnosis of sarcopenia.

Muscle mass—or more precisely lean soft tissue mass—remains an important indicator not only as part of the definition for sarcopenia but also cachexia and malnutrition. To raise broader awareness of the importance of muscle mass and function for patients’ physical ability, simple clinical surrogate markers could be used to estimate muscle mass [9]. Different studies pointed out that calf circumference (CC) shows a significant correlation with skeletal muscle mass (SMM) [10–12]. Furthermore, the age-related decrease in calf muscle appears to be more pronounced compared to mean arm circumference. Thus, CC not only has the potential to be an indicator of muscle mass, but also represents an anthropometric method to generate non-invasive, and easily reproducible measurements at low costs [13, 14]. Although anthropometry is not ideal for detecting a reduction of muscle mass, CC has been found to strongly correlate with fat free mass [13, 15], frailty and functional capacity [10, 11, 16]. The EWGSOP2 guidelines suggest a CC <31 cm as a proxy for low muscle mass in settings where no other diagnostic methods are available [1]. The AWGS include low CC as a case finding indicator with cut-off values of 34 cm for men and 33 cm for women [4]. There is growing evidence that CC is a useful measurement in the evaluation of low muscle mass in Asian countries [12, 17]. However, there is lack of data from European countries and the growing age group of individuals over 75 years. Ethnicity and environmental effects such as diet patterns and physical activity need to be considered as they influence body composition. In addition, gender differences need to be taken into account for the definition of a cut-off point, as men have significantly more muscle mass than women [14].

The aim of this study was to investigate the correlation between CC and appendicular skeletal muscle mass index (ASMI) among geriatric inpatients of a University Hospital Department of Geriatric Medicine, to evaluate the validity of the suggested CC cut-off point in the EWGSOP2 guidelines, and to subsequently determine relevant CC-cut-off values in order to simplify the determination of low muscle mass in clinical practice.

## Methods

### Study design and population

This cross-sectional single center study is part of a previous study, assessing the prevalence of sarcopenia in hospitalized geriatric patients using the EWGSOP2 guidelines. These results and associated parameters have been published previously [18]. The study was conducted in Switzerland and included 305 consecutively recruited patients admitted to acute geriatrics and geriatric rehabilitation between September 10 and October 30, 2019. Patients with acute sepsis, severe dehydration or volume overload, short life expectancy, factors affecting measurements with BIA, and inability to follow study procedures were excluded. Informed consent was obtained from all subjects or their legal representative. All methods were performed in accordance with relevant guidelines and regulations.

The study was conducted in accordance with the Declaration of Helsinki. All experimental protocols were approved by the Ethical Review Committee of Northwest and Central Switzerland (BASEC ID 2019-01461, 28/08/2019) and was registered at ClinicalTrials.gov (NCT04124575, 11/10/2019).

### Data collection

All participants were assessed within the first six days of hospital admission. Age and gender, length of hospital stay, comorbidities, and number of drugs at hospital admission, results of the baseline geriatric examination (mini mental state exam, timed up and go test, nutritional risk screening, functional independence measure) were extracted from medical records. Body height (cm), weight (kg), calf and mid-arm circumference (cm) were measured using standard methods.

### Assessment of sarcopenia

Sarcopenia was diagnosed based on the EWGSOP2 definition and cut-off values [1]. In this highly vulnerable population, all patients were assessed based on clinical suspicion. To assess muscle strength, a pneumatic hand dynamometer (Martin Vigorimeter^®^, Gebrueder Martin GmbH, Tuttlingen, Germany) was used. Cut-off values for low handgrip strength were <50 kPa for men and <34 kPa for women >75 years old, and <64 kPa for men and <42 kPa for women ≤75 years old [19]. To determine muscle mass, BIA was performed (tetrapolar whole-body device BIA 101, Akern, Florence, Italy). All participants were assessed in supine position with extremities stretched. The estimates obtained for the evaluation of appendicular skeletal muscle mass (ASMM) were derived from proprietary manufacturer algorithms and using Bodygram Plus software, version 1.2.2.8 (Akern, Florence, Italy). Cut-off values for low ASMI, calculated from ASMM/height^2^, were <7.0 kg/m^2^ for men and <5.5 kg/m^2^ for women [1]. According to EWGSOP2 guidelines, sarcopenia was defined as probable when handgrip strength was low; confirmed when both handgrip strength and muscle quantity were low, and sarcopenia was defined as severe by additional documentation of low physical performance (timed up and go test ≥20 seconds).

### Measurement of calf circumference

Two trained health professionals performed the CC measurements. Measurements were taken once by one of the two examiners. Calf circumference was assessed in sitting or supine position [20]. For individuals in sitting position, measurements were taken on a chair in 90°-knee flexion with feet resting on the floor or on the footrest in a wheelchair. Subjects with a small stature were asked to sit on the bed and its height was adjusted to allow a 90° knee flexion. In the supine position, the knee joint was flexed at 90° with the feet and ankles relaxed. The flexible tape was wrapped perpendicular around the leg axis without pressing the tissue. Measurement was taken at maximum circumference and recorded at the nearest 0.5 cm. To confirm the point of greatest circumference, additional measurements were taken above and below the original measuring point to identify the maximal girth. At least three measurements were taken, and the maximum circumference was recorded. All measurements were taken on the right side unless there was previous amputation, acute fracture, wounds or bandages, or visual aspect of a weaker calf on the right leg. Compression stockings, if present were taken off just before the measurements. Patients presenting with non-removable plasters or bandages, volume overload or with pitting edema were excluded, according the exclusion criteria described above.

### Statistical analysis

Continuous variables were summarized as mean and standard deviation (SD). Categorical variables were presented as the absolute frequencies (n) and percentages (%). Linear regression analysis was used to evaluate CC and sex as predictors for ASMI.

Receiver operating characteristic curves (ROC) were used to calculate the discriminatory value of the CC cut-off values to separate patients above and below ASMI cut-offs for sarcopenia (<5.5 kg/m^2^ for women, <7.0 kg/m^2^ for men). Because sex is a known predictor of muscle mass, a sex-specific analysis was performed.

Statistical calculations were carried out with SPSS Version V22 (IBM SPSS Statistics, Chicago, IL) and R 3.6.3 (R Foundation for Statistical Computing, Vienna, Austria).

## Results

Out of 414 patients admitted, 29 were excluded due to acute sepsis, severe dehydration or volume overload, 13 had a short life expectancy, 12 did not wanted to participate, and 18 were excluded for other reasons. In addition, 37 participants were excluded, as they were assessed twice due to their admission to acute geriatrics prior to rehabilitation, resulting in a final study population of 305 patients (Table 1). Complete baseline patient characteristics and measurements are presented in a previously published study by Bertschi et al. [19]. Summarising, mean age was 83.5 (SD 7.0) years and 65.6% were female. The prevalence of sarcopenia was found to be 22.6%, of which most fulfilled the criteria for severe sarcopenia.

**Table 1:** Characteristics and measurements of the study population.

Characteristics	All n = 305	Males n = 105	Females N = 200
Age, years	83.5 (7.0)	82.9 (7.2)	83.8 (7.0)
Body mass index, kg/m^2^	25.7 (4.7)	26.1 (4.0)	25.5 (5.0)
Calf circumference, cm	32.6 (4.1)	33.2 (3.7)	32.4 (4.2)
Mid-arm circumference, cm	26.6 (4.0)	26.8 (3.7)	26.4 (4.2)
Handgrip strength, kPa	41.0 (14.5)	50.8 (16.1)	35.6 (10.4)^a^
ASMI, kg/m^2^	6.2 (0.9)	6.9 (0.8)	5.9 (0.8)
No sarcopenia Probable sarcopenia Confirmed sarcopenia	161 (52.8)	58 (55.2)	103 (51.5)
	75 (24.6)	19 (18.1)	56 (28.0)
	69 (22.6)	28 (26.7)	41 (20.5)

Values are presented as mean ± standard deviation for continuous variable or number and percent for dichotomous variables

*kPa* kilo Pascal, *ASMI* appendicular skeletal muscle mass index

^a^Significant group difference (p < 0.05) between males and females

**Fig. 1:**
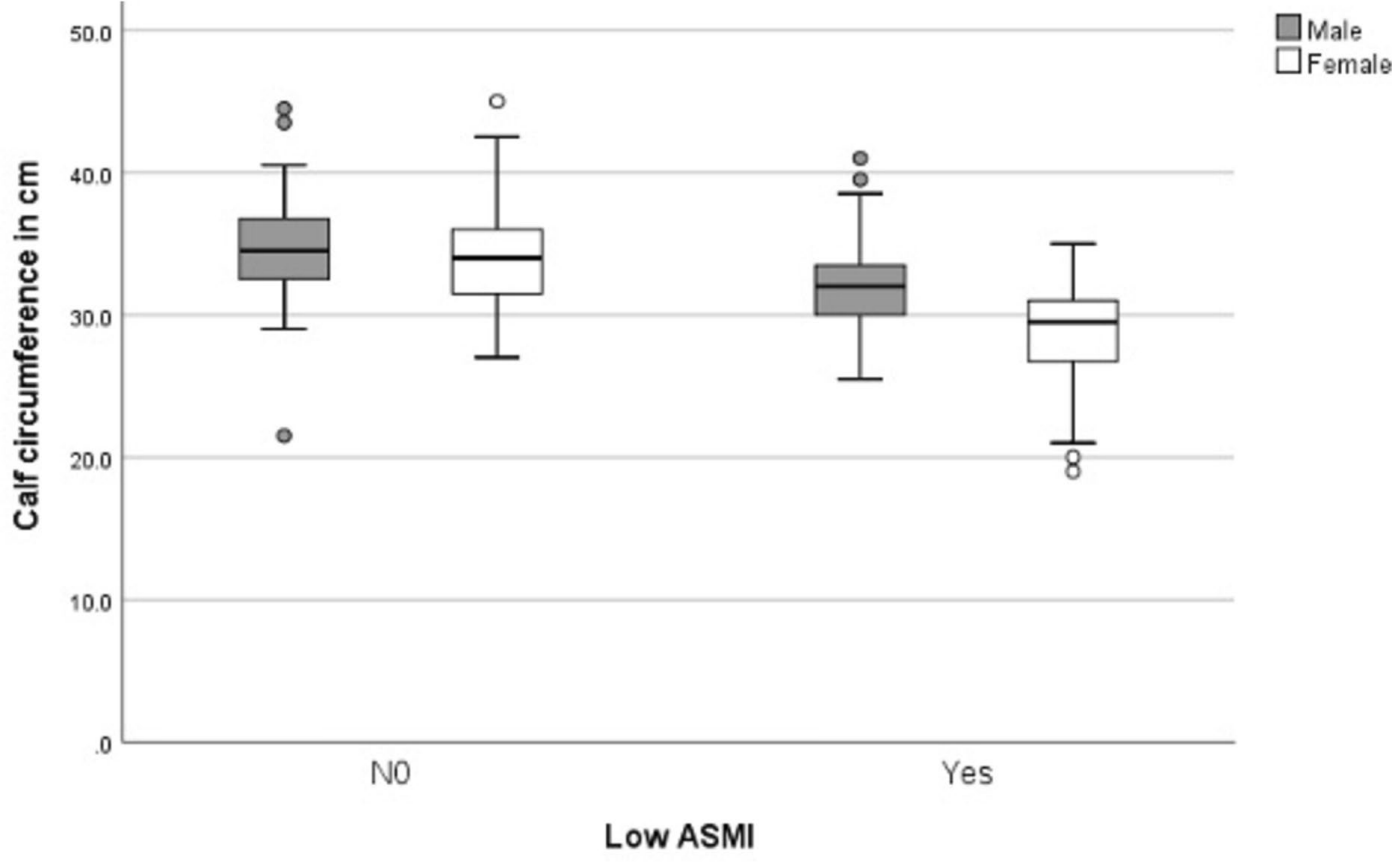
Box and whisker plot and five-number summaries of calf circumference. In males (n = 54) and females (n = 137) with normal appendicular muscle mass index (ASMI), and with low ASMI in males (n = 51) and females (n = 63)

**Fig. 2:**
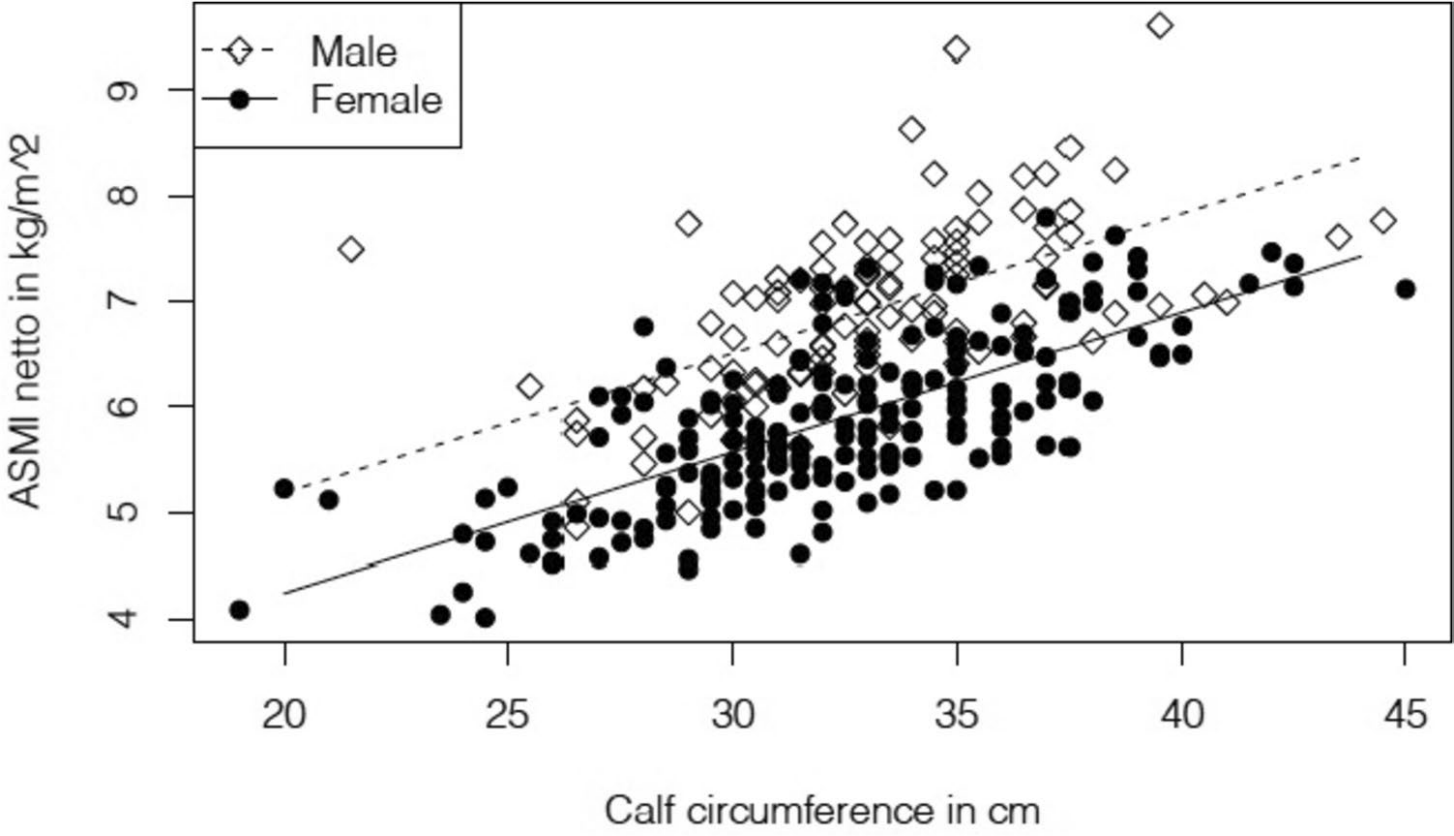
Regression analysis of calf circumference and appendicular skeletal muscle mass index (ASMI). In females (n = 200, circles) and males (n = 105, squares) The corresponding regression equations are for males 2.23 + 0.1324 * calf circumference and for females 1.6 + 0.1324 * calf circumference. R^2^ = .60, F(302) = 15.5 (p< 0001), and for difference between sex F(302) = − 12.98 (p<0.001)

**Fig. 3:**
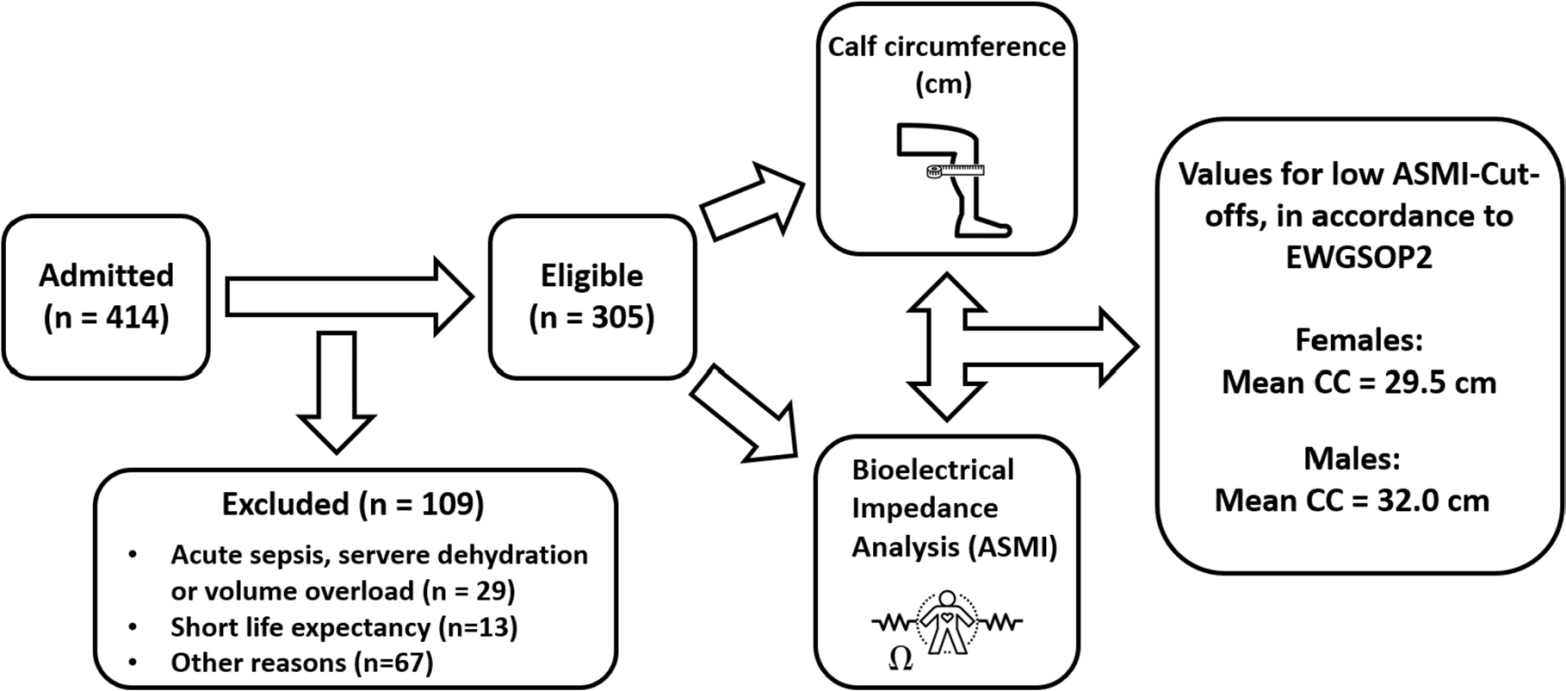
The flowchart illustrates the research process to investigate the relationship between Calf Circumference (CC) and muscle mass in geriatric patients. The study shows a significant correlation and establishes for the first time gender-specific CC cutoffs to improve sarcopenia assessment. *ASMI* appendicular skeletal muscle mass index, *cm* centimeter

The prevalence of low ASMI was 38.3%, it was lower for females than for males. In subjects with low ASMI, mean CC was 29.5 ± 3.4 cm for females and 32.0 ± 3.4 cm for males (Table 2)*.* Subjects with low ASMI had lower CC than those with ASMI above the sex-specific cut-off points defined in EWGSOP2 (Fig 1). The linear regression analysis showed a positive relationship between CC and ASMI. Sixty percent of the variation in ASMI can be explained by the model containing only CC (p <0.001) (Fig 2). The flowchart illustrates the research process to investigate the relationship between CC and established in geriatric patients (Fig. 3).

**Table 2:** Calf circumference at normal and low appendicular skeletal muscle mass index

	Normal ASMI	Low ASMI
Females, n (%)	137 (67.5)	63 (31.5)
Calf circumference cm, mean (SD)	34.0 (3.5)	29.5 (3.4)
Males; n (%)	54 (51.4)	51 (48.6)
Calf circumference, cm, mean (SD)	34.5 (3.6)	32.0 (3.4)

Values are presented as number and percent for dichotomous variables or mean ± standard deviation for continuous variable

*ASMI* Appendicular skeletal muscle mass index

ROC analysis was performed to confirm the criterion-related validity of CC for low muscle mass according to the suggested cut-off value of <31 cm by EWGSOP2 [4]. For women, this cut-off resulted in a sensitivity of 71% and a specificity of 84%. When the same cut-off was applied for men, sensitivity was 37%, and specificity 92%. Applying the AWGS cut-off values (33 cm female; 34 cm for male) resulted in a sensitivity and specificity of 89% and 63% for females and in 76% and 55% for males, respectively.

**Fig. 4:**
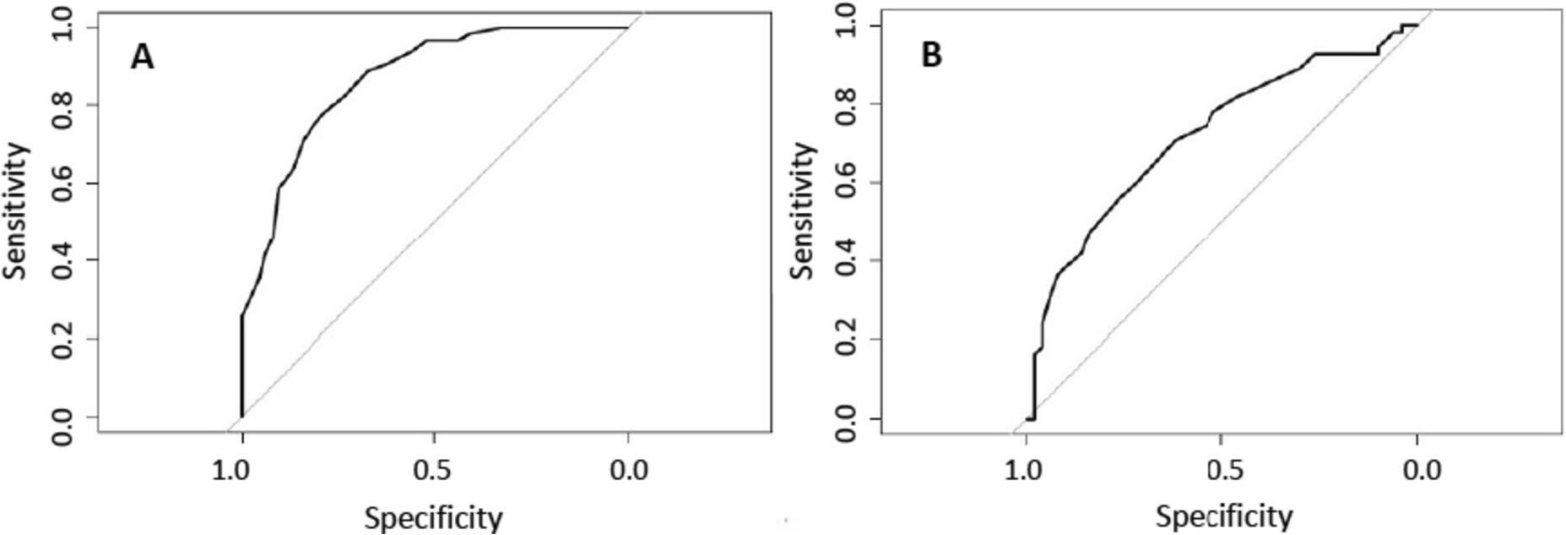
Receiver operating characteristic curves for predicting calf circumference for low appendicular muscle mass index. **A** In females (n = 200), the optimal cut-off for calf circumference was 31.5 cm (sensitivity 78%, specificity 79%), the area under the curve (AUC) was 0.87 (95% CI: 0.82–0.92). **B** In males (n = 105) the optimal cut-off was 33.5 cm (sensitivity 71%, specificity 62%) and AUC 0.72 (95% CI: 0.62–0.82)

The optimal cutoff value for CC from the ROC analysis, statistically defined as the best compromise between sensitivity and specificity for our group, was 31.5 cm for females (sensitivity 78%, specificity 79%), and 33.5 cm for males (sensitivity 71%, specificity 62%) (Fig. 4*).* These cutoffs were surpassed by 63 of 200 females and 55 of 105 males. The performance of these CC cut-offs for false positive and false negative results are displayed in Table 3.

**Table 3:** Performance of low calf circumference for low appendicular muscle mass index

	Females n = 200	Males n = 105
Sensitivity, n %	49 (78)	39 (71)
Specificity, n %	108 (79)	31 (62)
False positive, n (%)	29 (37)	19 (33)
False negative, n (%)	14 (11)	16 (34)

Cut-off points for low calf circumference for females < 31.5 cm, males < 33.5 cm and for muscle mass females < 5.5 kg/m^2^, males <7.0 kg/m^2^. Totals are given in Table 2 and in the text

## Discussion

The aim of this study was to 1) correlate ASMI with CC, 2) evaluate CC as a surrogate parameter and prognostic value of low muscle mass, and 3) critically question the validity of the current CC cut-off presented in the EWGSOP2 guidelines. We found a positive correlation between CC and muscle mass in our geriatric inpatient study population and were also able to establish, for the first time, important gender-specific CC cutoffs of 29.5 cm for women and 32.0 cm for men.

Although the radiological modalities for assessing muscle mass are convincing in terms of accuracy compared with CC determination, CC measurement in the elderly additionally impresses with its valuable predictability of fall events, frailty, malnutrition, morbidity and mortality [16, 21–25]**.** The anthropometric method for recording CC provides a viable and cost-effective option for diagnosing “confirmed sarcopenia” in the elderly according to the diagnostic algorithm of the EWGSOP-2 guidelines. Compared to advanced imaging modalities such as CT or MRI, CC measurement is easily accessible and does not require highly specialized equipment or trained personnel.

Consideration of gender-specific CC cutoffs is critical due to the significant muscular differences between men and women. Although EWGSOP2 guidelines already consider gender specific ASMM cutoffs, these differences have not yet been extended to anthropometric measures such as CC. It is undeniable that men and women have structural differences in their muscle mass that should be considered in an appropriate diagnostic context. The integration of gender-specific CC cutoffs allows for a more accurate and tailored gender diagnosis of sarcopenia, making the assessment of muscle health better adapted to individual biological differences between the genders and leading to more accurate results and, in our case, could also lead to earlier therapies and interventions.

Our results are in accordance with anthropometric reference data for elderly Swedes derived from 3360 subjects (60 – 99 years) where the 10^th^ percentile was 32 cm for women and 33.3 cm for men, respectively [26]. Another large cross-sectional study including non-hispanic white Americans (n = 8309) generated cut-off values using one or two standard deviations below the mean to define moderately or severely low CC values [27]. Cut-off values for moderately and severely low CC were 34.4 cm and 32.2 for males, and 33.4 cm and 31.2 cm for females, respectively.

In a French sample, Rolland et al. studied 1458 women who lived at home and were over 70 years of age [10]. Muscle mass was assessed via DXA and subsequently correlated with CC. They calculated a CC cut-off of 31 cm for the presence of sarcopenia. While the specificity was very high, the sensitivity of 44.3% was quite modest, so that many women may have been ignored with regard to the clinical picture. If the same cut-off had also been applied to male subjects, the sensitivity would have been even lower.

A Swedish working group around Sobestiansky et. al investigated the ratio of CC to total body muscle mass, which was calculated via DXA, on the basis of geriatric inpatients [28]. Here, a gender-independent cut-off of <31 cm for the presence of sarcopenia was determined and evaluated as an acceptable alternative to DXA as an indicator of low muscle mass. Although differentiation by gender would not have significantly altered the results with respect to CC, the present sample size of just 56 patients may have had limited representativeness for this specific question.

The EWGSOP2 guidelines provide a uniform, gender-independent CC cut-off value of <31 cm. However, a study by Asai et al. was able to investigate that there are gender-related differences with regard to muscle strength and, in particular, calf muscle mass, that are necessary for the determination of cut-off values for differentiation [29]. On the basis of 124 older participants, they were able to establish different strong correlations between the CC and the calf muscle mass obtained via MRI for women and men. Furthermore, using cadaveric dissections, Tresignie et al. were able to determine strong correlation for males and females in terms of CC and whole body tissue mass by examining 9 male and 14 female subjects [30]. They compared different body circumferences and related them to the respective whole body tissue mass. Again, these results support our hypothesis to consider gender differences to estimate muscle mass for future measurements. A study by Mienche et al. established CC cut-off values of <34 cm for sarcopenic men and <29 cm for sarcopenic women based on a cross-sectional study of 120 Asian patients over 60 years of age [31]. However, this study used the diagnostic criteria from AWGS which applied culturally different cut-off values and ASM was measured by DXA.

Chung-Yao-Chen et al. investigated CC cut-off values for sarcopenic individuals > 65 years by subsequently correlating ASM estimated by BIA with the CC in 177 participants [32]. They calculated a CC cut-off of <34 cm for men and <33 cm for women. With regard to our results, the values for men were only minimally higher in contrast of those for women. However, this study included elderly ethnic Chinese in assisted living and therefore direct comparability to our results is likely to be limited. In addition, the number of participants was significant smaller compared to ours. Nevertheless, these results, which reflect the recommendations of the AWGS, highlight the need for a gender-specific approach to CC in sarcopenic patients. The sensitivity and specificity for the suggested cut-off values where higher in women than in men, which is contrary to other findings, [11] and the fact that correlation of CC and ASM in men is generally higher than in women [33]. The smaller sample size of men as well as nonmuscular components of the calf in our sample of octogenarians might have contributed to this finding. Due to hormone changes, older men might experience changes in subcutaneous adipose tissue and intramusclular adipose tissue not detected by the circumference measurements. In general, correlations of muscle mass and CC was found to be smaller in older age, when compared with participants aged below 60 years [33].

Overall, measurement of CC proved to be a reliable and reproducible method, mainly because of small differences in measurement between different investigators [29]. However, intra- and extra-rater reliability was not assessed in this study. In addition, when using CC in the elderly and especially in a hospital setting, variations due to leg edema must be taken into account. After all, edema occurs in approximately 25% of hospitalized patients and can have a variety of causes [34]. One of the most common may be hyperhydration in the setting of right-sided or globally decompensated heart failure, often with concomitant renal insufficiency. Other causes would be postoperative lymphatic drainage disorders after hip osteosynthesis or inflammatory processes, which can be observed in erysipelas, for example. Thrombotic components such as deep vein thrombosis or its late sequelae, the postthrombotic syndrome, can also influence CC measurement results. This fact was demonstrated by Ishida et al. in a scientific review, who demonstrated that the presence of edematous leg swelling increases CC by an average of 2 cm in men and 1.6 cm in women leading to a misdiagnosis of 10% regarding the occurrence of sarcopenia [35]. Although we excluded patients with severe fluid overload in the present study, this is a clinical examination that is subjective and likely to show corresponding variability between different investigators.

In addition to the circumstances of edematous leg swelling already discussed, the use of measurement in supine and sitting position and the missing data on repetability of the CC measurement, the present study has other limitations. First, BIA tends to overestimate skeletal muscle mass compared to results obtained via DXA and is also more susceptible to a patient's hydration status [17]. Furthermore, sarcopenic, obese patients will likely not be correctly identified with CC measurement. Because there is no consensus to date on the definition of sarcopenia in such patients and although the mean BMI in our sample was 25.7 kg/m^2^, our cut-off values refer to non-obese, elderly patients. Moreover, the results of our study are particularly related to a majority female population and, besides, are not easily transferable to patients in other life situations, age groups, or ethnicities. Last, a major limitation of the present study was the fact, that only one method of muscle quantification was applied. To verify valid results of CC, different methods of quantitative assessment of muscle mass should be included in further studies to discuss the relationship between anthropometric data and functional performance measures using multivariate data analysis. This will allow to verify relevant CC cut-off values as a surrogate marker of low muscle mass and to gain additional insight into the complex relationship between muscle loss and physical function.

## Conclusion

This study offers valuable insights into sarcopenia assessment among elderly individuals. Our findings reveal a noteworthy correlation between CC and total body muscle mass, further enhanced by the establishment of gender-specific CC cutoffs, a novel contribution. These gender-specific thresholds provide a more precise diagnostic approach that accommodates the inherent muscular disparities between men and women, potentially facilitating early intervention strategies.

In addition to its precision, the anthropometric CC measurement stands out for its practicality, cost-effectiveness, and predictive capacity regarding fall risk, frailty, malnutrition, and morbidity, making it a compelling choice when compared to the accuracy of radiological methods.

We advocate for continued research to explore the generalizability of these outcomes across diverse populations and clinical settings. CC holds promise for advancing the early identification and management of sarcopenia, thereby enhancing the overall quality of life for elderly individuals.

## Data Availability

The datasets used and analyzed during the current study are available from the corresponding author on reasonable request.
